# *LEP* (G2548A-G19A) and *ADIPOQ* (T45G-G276T) gene polymorphisms are associated with markers for metabolic syndrome

**DOI:** 10.1186/s13098-023-01215-6

**Published:** 2023-11-17

**Authors:** Fred Luque Ortega, Alejandra Martínez Camberos, Martín Irigoyen Arredondo, Noemí García Magallanes, Eliakym Arámbula Meraz

**Affiliations:** 1 Laboratorio de Ciencias Básicas, Facultad de Odontología, Universidad Autónoma de Sinaloa, Culiacán Rosales, México; 2 Licenciatura en Ciencias Biomédicas, Universidad Autónoma de Occidente, Mazatlán, Sinaloa, 82100 México; 3 Laboratorio de Biomedicina y Biología Molecular, Unidad Académica de Ingeniería en Biotecnología, Universidad Politécnica de Sinaloa, Mazatlán, Sinaloa, 82199 México; 4 Laboratorio de Genética y Biología Molecular, Facultad de Ciencias Químico-Biológicas, Universidad Autónoma de Sinaloa, Ciudad Universitaria, Av de las américas, Mexico City, México

**Keywords:** Metabolic syndrome, LEP, ADIPOQ, Haplotype

## Abstract

**Background and aims:**

There is a link between genetics with metabolic balance and adiposity homeostasis on metabolic syndrome (MetS). Polymorphism in adipokine genes such as leptin and adiponectin may play an important role in its development. This study aimed to determine the association of the individual and general components of MetS with genetic alterations in *LEP* (rs7799039 and rs2167270) and *ADIPOQ* (rs1501299 and rs2241766) genes in the Mexican population.

**Methods and results:**

The polymorphisms of the *LEP* gene rs7799039 and rs2167270, together with rs1501299 and rs2241766 polymorphisms of the *ADIPOQ* gene were genotyped using polymerase chain reaction-restriction fragment length polymorphism (PCR-RFLP) on 328 individuals (n = 131 MetS). The rs7799039 under the recessive inheritance model was found to be associated with increased risk of MetS (OR = 2.16, 95% CI = 1.06–4.37), dyslipidemia (OR = 7.97, 95% CI = 2.17–29.36), low HDL (OR = 7.01, 95% CI = 1.65–29.71) and hypertension (OR = 13.02, 95% CI = 1.76–96.44); the heterozygote demonstrate a protective effect on MetS (OR = 0.48, 95% CI = 0.28–0.88) and diabetes (OR = 0.09, 95% CI = 0.02–0.53) under the over the dominant model. Haplotype analysis showed linkage disequilibrium between the SNPs of *ADIPOQ* rs1501299/rs2241766, and their association as risk factors for low HDL and hypertension.

**Conclusion:**

The association of rs7799039 with the presence of MetS, suggests a risk factor for the development of dyslipidemia, as well as its heterozygous as a protective factor for DM. There is a linkage disequilibrium between the SNPs of *ADIPOQ.*

**Supplementary Information:**

The online version contains supplementary material available at 10.1186/s13098-023-01215-6.

## Introduction

Metabolic Syndrome (MetS) is an increasing public health problem worldwide. It is a cluster of disorders that together lead to progressive disease with five times more vulnerable to developing diabetes mellitus type 2 (DM2) and 2.5 times more likely to die from cardiovascular diseases [1, 2]. Among the indicators of MetS are abdominal obesity, atherogenic dyslipidemia, elevated blood pressure, and hyperglycemia [3], considering central obesity as a starting point in most cases [4].

The development of obesity is usually the result of a chronic imbalance between energy intake and energy expenditure [5], however, this does not evaluate the metabolic alterations that regulate energy balance, such as the peptide hormone leptin, secreted primarily from white adipose tissue into the bloodstream to bind its receptors in peripheral tissues, inducing gluconeogenesis in the liver and inhibits the release of insulin from pancreatic β cells; it has been proved that insufficient leptin action leads not only to the development of obesity but also increases insulin resistance [6, 7]. Several single-nucleotide polymorphisms (SNPs) found in the *LEP* (Leptin) gene may be associated with serum leptin concentration related to the pathophysiology of obesity and its complications among specific ethnic groups [8].

Another relevant protein secreted exclusively by adipocytes to regulate metabolic processes is adiponectin (encoded by the ADIPOQ gene) [9]. Adiponectin is recognized as having anti-inflammatory, insulin-sensitizing, and anti-atherogenic properties and plays a critical role in the development of insulin resistance [10]. Numerous SNPs are associated with the pathophysiology of MetS, obesity, and associated complications [2, 11]. The polymorphisms rs1501299 and rs2241766 may act through decreased adiponectin expression, which may in turn cause increased body weight and insulin resistance [10, 11].

The SNPs in this study were chosen on the basis that previous studies have shown varying contrasting associations with MetS and their components [8–10, 12]. The conflicting results point to potential inter-ethnic differences in genetic predisposition to the diseases [9]. The highest prevalence of MetS among Hispanic/Latino adults [3], aimed to evaluate the association of the individual and general components of MetS with genetic alterations in *LEP* (rs7799039 and rs2167270) and ADIPOQ (rs1501299 and rs2241766) genes in Mexican population.

## Methods

### Study population

A blood sample was taken by venipuncture from 328 fasting individuals submitted to Regional General Hospital #1 and Family Medicine Unit #36, belonging to the Mexican Institute of Social Security (IMSS). MetS group was classified according to the criteria of the Latin American Diabetes Association with its working group Latin American Group for the Study of MetS (ALAD/GLESMO), with an adjustment for the parameters of abdominal diameter for the diagnosis of obesity for the Latin American population suggested by the International Diabetes Federation (waist circumference (WC) men above 94 cm and 88 cm in the case of women); controls were determined to be clinically healthy, with no indicators of MetS. Clinicopathological characteristics such as age, gender, weight, blood pressure, and biochemical parameters were collected through direct questionnaires and serological tests. All patients were approved by signing an informed consent review and approved by the Local Committee for Research and Ethics in Health Research of the IMSS.

### Biochemical parameters

Overnight fasting blood samples were collected from each participant to measure serum lipids, total cholesterol (TC), triglycerides (TG), high-density lipoprotein (HDL), Low-density lipoprotein (LDL), total cholesterol (TC), fasting blood glucose (FBG) and leptin levels.

### Genotyping of single-nucleotide polymorphisms

Genomic DNA was extracted from peripheral blood leukocyte pellets using the alt precipitation method [13] and diluted to a working concentration of 20 ng/ml for further use and stored at -20°C until use for genotyping. The genotyping of polymorphism *LEP* G2548A (rs7799039) *LEP* G19A (rs2167270), ADIPOQ T45G (rs2241766), and ADIPOQ G276T (rs1501299) were performed by polymerase chain reaction-based Restriction Fragment Length Polymorphism (PCR-RFLP) methods. Amplification of DNA sample was done by using the following flanking primers for *LEP*: F: 5′- CTCTGGAGGGACATCAAGGA-3’and R: 5′- GCCAAGAAAGACCAGCAGAG-3’ and for *ADIPOQ*, F: 5′- TCTCTCCATGGCTGACAGTG-3’ and R: 5′- AGATGCAGCAAAGCCAAAGT-3’.

The PCR procedure was performed with 50µL reaction mixture (200ng of template DNA, 20µM of each primer, 0.2MM of dNTPs, 1 U/ml of Taq Polymerase, and 1X PCR buffer, consisting of an initial melting step of 10 min at 94° C followed by 30 cycles of 45 s at 94° C, 45 s at 60° C and final elongation step of 10 min at 72 °C.

PCR products (468 bp for *ADIPOQ* and 353 bp for LEP) were detected on 1.5% agarose gel stained with gel red; amplified PCR products were digested with 20U of restriction enzymes *SmaI* (*ADIPOQ* T45G), *BsmI* (*ADIPOQ* 5276T), *MspA1I* (LEP G19A) and *HhaI* (LEP G2548A) according to manufacturer instructions (BioLabs, New England). The restriction products were resolved by 7.5% polyacrylamide gel electrophoresis against 100 bp ladder as standard at 100 V for 1 h and stained with silver nitrate to visualize the products. For each reaction, a negative control (without a DNA template) was added to monitor PCR contamination. For *ADIPOQ*, the digestion resulted in fragments of 86 and 382 bp for the G allele (T45G), and 320 bp and 148 bp for the T allele (G27T); the homozygous wild allele and mutant, respectively, remain undigested. In LEP digestion products, the G allele (G19A) resulted in fragments of 59 and 294 bp, also 182 bp and 60 bp for the G allele (G2548A); the homozygous mutant allele, remains undigested in both SNPs.

### Statistical analysis

Statistical tests were performed using SPSS 20.0 (IBM corporation, Chicago, IL, USA). The quantitative data were represented as means ± SD and compared by independent sample t-test. Variables without normal distribution were compared through the Mann-Whitney U test.

Hardy–Weinberg equilibrium (HWE) and linkage disequilibrium (LD) for the polymorphisms were calculated with the use of SNPStats software (https://www.snpstats.net/start.htm)(14) and HaploView software. Multiple inheritance models were used to assess statistically significant associations between genotypes and MetS: codominant, dominant, recessive, and over-dominant. The qualitative data were expressed in percentages, which were further analyzed using the Chi-square test. Associations of clinical variables with polymorphisms were evaluated by logistic regression to obtain odds ratio (OR) and 95% confidence intervals (CI). To account for covariate effects on genotypes and haplotypes, statistics were adjusted for age, sex, and WC. A p-value less than 0.05 was considered statistically significant.

## Results

The anthropometric and clinical parameters of all the participants are presented in Table 1. The 36.6% of the study population were women, 32.5% of them with MetS. Individuals with MetS had higher age, WC, SBP, DBP, FBG, TG, TC, LDL, and leptin levels compared to levels in those without MetS (p < 0.05). Of those with WC above limits (94 cm and 88 cm in the case of women), 61.5% were MetS patients and 38.5% were controlled. 72.8% of patients with FBG > 100 mg/dL were classified with MetS. Of those with a serum TG level of ≥ 150 mg/dL, 82.5% were MetS patients. Low HDL was identified in 62% of MetS and 32% of control groups.

**Table 1:** Main characteristics of the metabolic syndrome (MetS) and control groups

Parameter	MetS (n = 131)	Control (n = 197)	p-value
Age (years old)	37.6 ± 12.4	31.8 ± 11.6	< 0.001
Gender (M/F)	92/39	116/81	0.037
WC (cm)	107.1 ± 10.5	91.5 ± 10.6	< 0.001
SBP	124.6 ± 14.5	115.1 ± 12.9	< 0.001
DBP^1^	85 ± 10.6	75.6 ± 9.8	0.003
FBG (mg/dL)	108.9 ± 38.9	90.3 ± 8.8	< 0.001
TG (mg/dL)	179.6 ± 78.5	96.6 ± 46.2	< 0.001
TC (mg/dL)^1^	187.9 ± 42.0	171.1 ± 35.3	< 0.001
HDL (mg/dL)	35.8 ± 8.4	46.6 ± 11.4	< 0.001
LDL (mg/dL)	115.8 ± 38.9	105.4 30.1	0.023
Leptin (ng/mL)	11.3 ± 11.7	7.2 ± 6.9	< 0.001

M: male, F: female; WC: waist circumference; SBP, systolic blood pressure; DBP: diastolic blood pressure; FBG: fasting blood glucose; TG: triglyceride; TC: total cholesterol; HDL: high-density lipoprotein; LDL: low-density lipoprotein

Values for continuous variables are expressed as the mean ± standard deviation (SD). ^1^ The t-test was used for those variables. The Mann–Whitney U test was utilized

In both MetS and control groups, genotype distribution and allele frequencies for *LEP* and *ADIPOQ* polymorphism have been presented in Table 2. The *LEP* G2548A and G19A polymorphisms were common among the MetS group; allele frequencies were similar between groups. In *ADIPOQ* T45G and G276T, the normal genotype had a higher frequency. There was no significant difference between the two groups according to genotype and allele frequencies of any SNP. The distribution of genotypes observed in both the control and case samples was in HWE.

**Table 2:** Genotype and allele frequencies of single nucleotide polymorphisms (SNPs).

Gene	SNP (rs)	Genotypes / Alleles	Control group % (n)	MetS group % (n)	p
***LEP***	G2548A (rs7799039)	GG	20.8 (40)	25.2 (33)	0.198
		GA	60.4 (116)	50.4 (66)	
		AA	18.8 (36)	24.4 (32)	
		G	51 (196)	50 (132)	0.896
		A	49 (188)	50 (130)	
	G19A (rs2167270)	GG	24.9 (49)	30 (39)	0.570
		GA	45.7 (90)	43.9 (57)	
		AA	29.4 (58)	26.1 (34)	
		G	52 (206)	48 (125)	0.292
		A	48 (188)	52 (135)	
***ADIPOQ***	T45G (rs2241766)	TT	69.5 (137)	66.4 (87)	0.403
		TG	28.4 (56)	29 (38)	
		GG	2 (4)	4.6 (6)	
		T	84 (330)	81 (212)	0.347
		G	16 (64)	19 (50)	
	G276T (rs1501299)	GG	44.2 (87)	50 (65)	0.574
		GT	46.2 (91)	40.8 (53)	
		TT	9.6 (19)	9.2 (12)	
		G	67 (265)	70 (183)	0.400
		T	33 (129)	30 (77)	

Chi-square test. Genotype and allele distributions of *LEP* and *ADIPOQ* variants were expected from the HWE (p > 0.05)

However, a significant difference was not observed in the allele frequency analysis, multiple inheritance models were used to assess associations between genotypes and MetS. The LEP G2548A showed statistically significant associations in three inheritance models after adjustments for age, gender, and WC: codominant (p = 0.037), recessive (p = 0.032), and overdominant (p = 0.017). However, in the codominant inheritance model, the A allele did not confer the risk of MetS. The rs2167270, rs2241766, and rs1501299 did not show statistically significant associations with MetS in all the inheritance models (Supplementary Table 1).

In the recessive model (Table 3), the − 2548AA homozygous genotype of *LEP* carriers had an increased risk for the development of dyslipidemia (OR = 7.97, 95% CI = 2.17–29.36; p = 0.008). The heterozygous shows a protective effect over DM (OR = 0.09, 95% CI = 0.02–0.53; p = 0.085) in the overdominant model.

**Table 3:** Association of *LEP* G2548A (rs7799039) with indicators of MetS

Indicator	Inheritance model							
	Recessive				Overdominant			
	GG-GA	AA			GG-AA	GA		
	OR (95% CI)^1^			p	OR (95% CI)^1^			p
Dyslipidemia	0.74 (0.32–1.71)	**7.97 (2.17–29.36)**		**0.008**	2.53 (0.85–7.46)	0.56 (0.22–1.43)		0.190
TG > 150 mg/dL	1.47 (0.74–2.94)	1.26 (0.37–4.26)		0.077	0.64 (0.25–1.62)	0.58 (0.26–1.31)		0.100
FBG > 100 mg/dL	1.36 (0.67–2.78)	1.78 (0.47–6.73)		0.420	0.93 (0.35–2.48)	0.59 (0.25–1.39)		0.480
Diabetes mellitus	0.37 (0.10–1.30)	3.41 (0.32–35.75)		0.230	1.15 (0.27–4.86)	**0.09 (0.02–0.53)**		0.085
Low HDL	1.53 (0.72–3.26)	**7.01 (1.65–29.71)**		0.320	2.41 (0.80–7.26)	0.76 (0.32–1.82)		0.640
Hypertension	1.24 (0.42–3.63)	**13.02 (1.76–96.44)**		0.120	3.03 (0.76–12.02)	0.63 (0.17–2.34)		0.340
Obesity	1.03 (0.50.2.13)	1.67 (0.64–4.38)		0.260	0.45 (0.18–1.13)	0.31 (0.12–0.77)		0.061

^1^Multiple logistic regression analysis was performed with adjustment for age, sex, and waist circumference

**Fig. 1:**
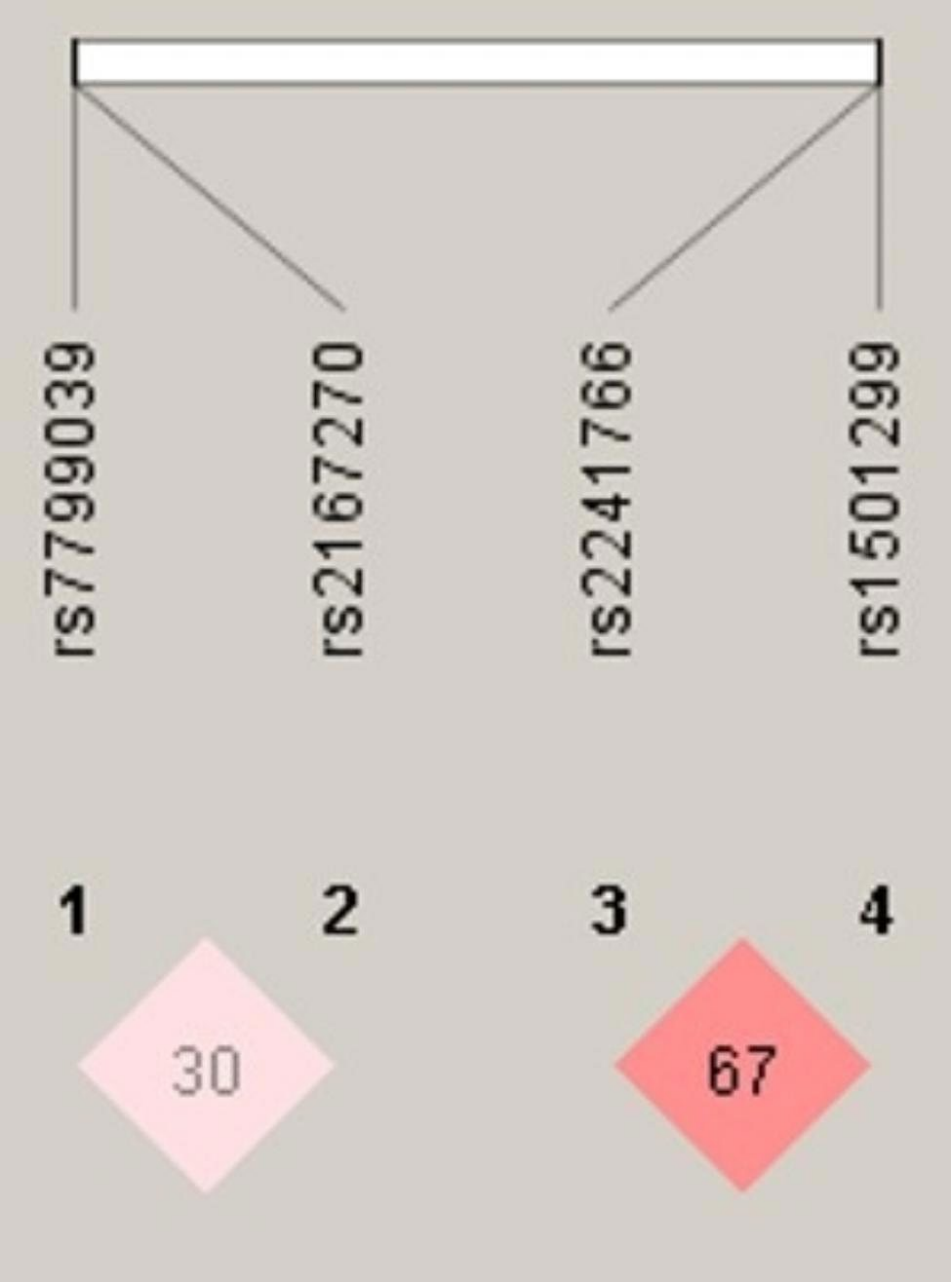
Linkage disequilibrium plot showing the position of LEP (rs7799039 and rs2167270) and ADIPOQ (rs2241766 and rs1501299) polymorphism and pair-wise D’ values observed in the study population concerning MetS; analysis by HaploView software

Based on the measures LD, it could be inferred that the two studied SNPs of *ADIPOQ* (rs2241766 and rs1501299) were in moderate LD (D´0.6741, r^2^ 0.044, p < 0.001) among MetS risk (Fig. 1). The global frequencies of haplotypes with different combinations of polymorphisms (*ADIPOQ* T45G/G276T) between MetS and control groups are shown in Table 4. Association of the haplotypes with covariates indicates a higher susceptibility to low HDL (OR = 4.37, 95% CI = 1.10-17.33) and hypertension (OR = 8.73, 95% CI = 1.12–67.84) in the carriers of GG haplotype; thus, the GT haplotype consisting of two mutated alleles-45G and − 276T, confer a protective effect towards the obesity (Table 5).

**Table 4:** *ADIPOQ* haplotype association with MetS

Haplotype (T45G/G276T)	Control	MetS	OR (CI 95%)	p	OR (CI 95%)^1^	p^1^
	Frequency %					
TG	53.5	52.9	1	-	1	-
GG	13.8	17.6	0.94 (0.65–1.38)	0.770	1.08 (0.64–1.84)	0.770
GT	2.5	1.2	1.31 (0.80–2.12)	0.280	1.88 (0.91–3.89)	0.089
TT	30.2	28.3	0.50 (0.11–2.37)	0.390	0.66 (0.10–4.40)	0.670

^1^Multiple logistic regression analysis was performed with adjustment for age, sex, and waist circumference

**Table 5:** *ADIPOQ* haplotype association with indicators of MetS

Indicator	Haplotype (T45G/G276T)				
	TG	GG	GT	TT	
	OR (CI 95%)^1^				p^1^
Dyslipidemia	0.75 (0.21–2.72)	1.36 (0.40–4.62)	-	1.22 (0.49.3.07)	0.750
TG > 150 mg/dL	1.27 (0.42–3.82)	0.98 (0.28–3.43)	-	1.33 (0.62–2.84)	0.360
FBG > 100 mg/dL	1.46 (0.50–4.30)	1.46 (0.37–5.83)	-	1.39 (0.62–3.11)	0.240
Diabetes mellitus	0.39 (0.08–2.04)	0.75 (0.17–3.37)	-	0.57 (0.08–3.85)	0.530
Low HDL	1.99 (0.61–6.50)	**4.37 (1.10-17.33)**	-	1.35 (0.54–3.41)	0.110
Hypertension	1.74 (0.31–9.69)	**8.73 (1.12–67.84)**	-	0.62 (0.14–2.70)	0.180
Obesity	1.35 (0.43–4.29)	1.10 (0.36–3.42)	**0.01 (0.001–0.03)**	0.94 (0.41–2.15)	0.590

TG: triglyceride; FBG: fasting blood glucose DM: Diabetes mellitus; HDL: high-density lipoprotein

^1^Multiple logistic regression analysis was performed with adjustment for age, sex, and waist circumference

## Discussion

The prevalence of MetS varies, greatly depending on the definition used, gender, age, socioeconomic status, and ethnic background [15]. Thus, the present study aimed to estimate the prevalence of the individual and general components of metabolic syndrome, considering sex-related differences, and assess the impact of age, sex, BMI, education level, and physical activity on the likelihood that participants present the metabolic syndrome. Genetic predisposition can have a great impact on the development of this syndrome, a higher prevalence of MetS has been demonstrated among Hispanic/Latino adults [3].

LEP G2548A and G19A polymorphisms are common genetic variants in people with MetS. These polymorphisms are found in the *LEP* gene, which encodes a protein that controls appetite and weight regulation. The presence of these polymorphisms may contribute to leptin resistance and, therefore, to obesity and metabolic syndrome [8, 16, 17]. Based on the analysis of genotypic frequencies in the Mexican population, the most common genotype for *LEP* G2548A was the GA genotype in both study groups, which differs from the frequencies reported in other populations [8]. An interesting observation after adjustment for age, sex, and WC is that the G2548A variant was shown to be associated with MetS in three inheritance models (codominant, recessive, and overdominant) reported in this study in the Mexican population. This is in contradiction with the results of the previous study, in which a relationship between this *LEP* polymorphism and the risk of MetS was determined [8, 16]. Nevertheless, it is consistent with reports from other populations such as Taiwan and Brazil [18, 19]. The − 2548AA homozygous genotype of LEP carriers had an increased risk for the development of dyslipidemia and − 2548GA shows a protective effect on DM, the biological causes of this are unknown, however, it is known that overdominant models occur when individuals heterozygous for a locus show a higher biological efficacy than individuals homozygous for that same locus, where the most severe manifestation for the disease or health status is when a double dose is received, either normal homozygous or mutated homozygous.

On the other hand, the GA genotype of the *LEP* G19A polymorphism was also frequently observed in both study groups which agrees with the report in the North Indian Punjabi population [20] and differs from the previous report we found in the literature performed in Egyptian population. In this report conducted in 2020, they describe that this polymorphism is highly associated with obesity and theorize that although it is located in a non-coding region, the association is attributed to its critical position in the 5’UTR regulatory region [20].

Because *ADIPOQ* regulates metabolism and lipid levels, the potential associations between *ADIPOQ* SNPs and MetS were also investigated. The *ADIPOQ* gene provides instructions for making a protein called adiponectin, which regulates glucose levels and fatty acid breakdown in the body. T45G and G276T have been associated with various health outcomes, including obesity, insulin resistance, and type 2 diabetes [21]. Concerning the *ADIPOQ* T45G and G276T variants in our study, the normal genotype had a higher prevalence among patients in both study groups, and no statistically significant evidence was found that the distribution of genotype frequency was different between the two study groups. This is not to the reports in the Asian population published by Kaur et al., where the most common genotype for the G276T variant was the mutated TT [11]. In the case of the T45G variant, no conclusive information was found on the distribution of genotypes in populations. However, some studies report that the mutated T allele was the most frequent concerning the G allele, which supports our results, where the T allele was the most common in our study populations in both groups [22]. In the *ADIPOQ* polymorphic variants we are reporting, they were not observed to be associated with MetS, despite adjustment for age, sex, and WC. However, when performing the haplotype analysis, we identified a moderate probability of LD, which is why it was interesting to analyze further detail. The combination in the “*ADIPOQ* T45G/G276T” haplotype could influence the production, secretion, and function of adiponectin, which in turn could have implications for the risk of developing metabolic syndrome by association with its indicators [23]. In our results, despite differences in the CI for the covariates of hypertension, obesity, and low HDL, such a statistical association was not verified. However, reported ADIPOQ haplotypes that include the T45G and G276T variations, associated with indicators of the MetS, such as type 2 diabetes and obesity, also link their findings with risk factors associated with the disease such as FBG, BMI, and lipid parameters, which suggests its crucial role in susceptibility to metabolic disease [24]. Further research is needed to understand the clinical aspects of genetic variants regulating adiponectin levels in other cohorts.

The frequency distributions of these polymorphisms differ between populations and, therefore, the susceptibility of the individual to the diseases related to these variations. These divergent results could be due to interactions of the polymorphisms with environmental factors, as well as with other polymorphisms present in the LEP and/or *ADIPOQ* genes, the sample size of a population, or the model used in the statistical analysis.

## Conclusion

In conclusion, the present work demonstrated the association of the *LEP* G2548A variant with the presence of MetS, suggesting a risk factor as part of the development and implications of MetS. Likewise, the homozygous *LEP* − 2548AA genotype was observed as a risk factor for the development of dyslipidemia, as well as the heterozygous *LEP* − 2548GA genotype as a protective factor for DM in the Mexican population. Our findings suggest that variants in LEP are of utmost relevance for the understanding of MetS development.

### Electronic supplementary material

Below is the link to the electronic supplementary material.

Supplementary Material 1

## Data Availability

Clinicopathological data presented in this work was the only information available in the hospital database. Raw data supporting our findings were generated at the Universidad Autonoma de Sinaloa and the Universidad Politecnica de Sinaloa. Raw data is available if requested from the corresponding author.
