# Supplementary material for: LEP (G2548A-G19A) and ADIPOQ (T45G-G276T) gene polymorphisms are associated with markers for metabolic syndrome
Source: Diabetol Metab Syndr. 2023 Nov 17;15:237. doi: 10.1186/s13098-023-01215-6 (PMC10656912; doi:10.1186/s13098-023-01215-6)
Supplement: Supplementary file 1 — Supplementary Material 1 [file 13098_2023_1215_MOESM1_ESM.docx]

**Supplementary material**

Table S1. Association of inheritance models of rs7799039 (*LEP* G2548A), rs2167270 (*LEP* G19A), rs2241766, and rs1501299 (*ADIPOQ* T45G and G276T) with MetS.

| **Gene / SNP** | **Model** | **Genotype** | **Control group n (%)** | **MetS group n (%)** | **OR (95% CI)^1^** | **p^1^** |
| --- | --- | --- | --- | --- | --- | --- |
| *LEP* G2548A | Codominant | GG | 40 (20.8) | 33 (25.4) | 1 | **0.037** |
|  |  | GA | 116 (60.4) | 65 (50) | 0.59 (0.28-1.23) |  |
|  |  | AA | 36 (18.8) | 32 (24.6) | 1.51 (0.64-3.56) |  |
|  | Dominant | GG | 40 (20.8) | 33 (25.4) | 1 | 0.490 |
|  |  | GA-AA | 152 (79.2) | 97 (74.6) | 0.79 (0.40-1.56) |  |
|  | Recessive | GG-GA | 156 (81.2) | 98 (75.4) | 1 | **0.032** |
|  |  | AA | 36 (18.8) | 32 (24.6) | **2.16 (1.06-4.37)** |  |
|  | Overdominant | GG-AA | 76 (39.6) | 65 (50) | 1 | **0.017** |
|  |  | GA | 116 (60.4) | 65 (50) | **0.48 (0.28-0.88)** |  |
| *LEP*  G19A | Codominant | A/A | 58 (29.4) | 34 (26.4) | 1 | 0.480 |
|  |  | G/A | 90 (45.7) | 57 (44.2) | 1.10 (0.55-2.20) |  |
|  |  | G/G | 49 (24.9) | 38 (29.5) | 1.57 (0.73-3.38) |  |
|  | Dominant | A/A | 58 (29.4) | 34 (26.4) | 1 | 0.470 |
|  |  | G/A-G/G | 139 (70.6) | 95 (73.6) | 1.27 (0.67-2.39) |  |
|  | Recessive | A/A-G/A | 148 (75.1) | 91 (70.5) | 1 | 0.230 |
|  |  | G/G | 49 (24.9) | 38 (29.5) | 1.48 (0.77-2.85) |  |
|  | Overdominant | A/A-G/G | 107 (54.3) | 72 (55.8) | 1 | 0.690 |
|  |  | G/A | 90 (45.7) | 57 (44.2) | 0.89 (0.49-1.60) |  |
| *ADIPOQ*  T45G | Codominant | T/T | 137 (69.5) | 87 (66.9) | 1 | 0.270 |
|  |  | T/G | 56 (28.4) | 37 (28.5) | 1.41 (0.71-2.77) |  |
|  |  | G/G | 4 (2) | 6 (4.6) | 3.31 (0.61-18.10) |  |
|  | Dominant | T/T | 137 (69.5) | 87 (66.9) | 1 | 0.190 |
|  |  | T/G-G/G | 60 (30.5) | 43 (33.1) | 1.54 (0.81-2.96) |  |
|  | Recessive | T/T-T/G | 193 (98) | 124 (95.4) | 1 | 0.200 |
|  |  | G/G | 4 (2) | 6 (4.6) | 3.01 (0.56-16.08) |  |
|  | Overdominant | T/T-G/G | 141 (71.6) | 93 (71.5) | 1 | 0.400 |
|  |  | T/G | 56 (28.4) | 37 (28.5) | 1.33 (0.68-2.62) |  |
| *ADIPOQ*  G276T | Codominant | G/G | 87 (44.2) | 65 (50.4) | 1 | 0.530 |
|  |  | G/T | 91 (46.2) | 52 (40.3) | 0.73 (0.39-1.34) |  |
|  |  | T/T | 19 (9.6) | 12 (9.3) | 1.08 (0.38-3.09) |  |
|  | Dominant | G/G | 87 (44.2) | 65 (50.4) | 1 | 0.400 |
|  |  | G/T-T/T | 110 (55.8) | 64 (49.6) | 0.78 (0.43-1.39) |  |
|  | Recessive | G/G-G/T | 178 (90.4) | 117 (90.7) | 1 | 0.650 |
|  |  | T/T | 19 (9.6) | 12 (9.3) | 1.26 (0.46-3.46) |  |
|  | Overdominant | G/G-T/T | 106 (53.8) | 77 (59.7) | 1 | 0.270 |
|  |  | G/T | 91 (46.2) | 52 (40.3) | 0.72 (0.40-1.29) |  |

^1^Multiple logistic regression analysis was performed with adjustment for age, sex, and waist circumference.
